# Fetal hormonal programming of sex differences in depression: linking women's mental health with sex differences in the brain across the lifespan

**DOI:** 10.3389/fnins.2014.00247

**Published:** 2014-09-08

**Authors:** Jill M. Goldstein, Laura Holsen, Robert Handa, Stuart Tobet

**Affiliations:** ^1^Division of Women's Health, Departments of Psychiatry and Medicine, Connors Center for Women's Health and Gender Biology, Brigham and Women's HospitalBoston, MA, USA; ^2^Departments of Psychiatry and Medicine, Harvard Medical SchoolBoston, MA, USA; ^3^Division of Psychiatric Neuroscience, Department of Psychiatry, Massachusetts General HospitalBoston, MA, USA; ^4^Department of Basic Medical Sciences, University of Arizona College of MedicinePhoenix, AZ, USA; ^5^Department of Biomedical Sciences and School of Biomedical Engineering, College of Veterinary Medicine and Biomedical Sciences, Colorado State UniversityFort Collins, CO, USA

**Keywords:** major depression, sex differences, women's health, stress, mood, fetal programming

Women's health has traditionally been thought of in the realm of reproductive health, and that includes women's mental health (i.e., perinatal psychiatry). However, we now know there are significant sex differences in many chronic diseases, including brain disorders. Thus, understanding the causes of sex differences in disorders of the brain, within and outside of reproduction, is critical to understanding women's mental health and healthcare needs. In order to accomplish this, it is necessary for neuroscience to adopt a “sex-dependent” and/or “sex-specific” lens on investigations of the brain. In this review, we make the case for depression, which has among the largest sex differences in disorders of the brain.

Major depressive disorder (MDD) recently became the number one cause of disability worldwide (Murray and Lopez, 1997; Ustun et al., 2004; World Health Organization, 2012). Importantly, the incidence of MDD in women is twice that of men (Kessler, 2003; Kendler et al., 2006), and thus understanding its pathophysiology has widespread implications for attenuation and prevention of disease burden, particularly in women. Over 40 years of research implicate hormonal dysregulation underlying mood disorders (Board et al., 1956; Gibbons and McHugh, 1962; Coplan et al., 2000; Brouwer et al., 2005; Kurt et al., 2007; Barim et al., 2009), particularly involvement of hypothalamic-pituitary-adrenal (HPA) and HP-gonadal (HPG) axes (Board et al., 1956; Gibbons and McHugh, 1962; Plotsky et al., 1998; Young and Korszun, 2002; Swaab et al., 2005). Central dysregulation of hormonal axes can precede MDD onset suggesting a role for hormonal abnormalities in female MDD vulnerability. Ours and others' work demonstrated that the vulnerability for sex-dependent risk for MDD begins in *fetal* development (McClellan et al., 2010; Goldstein et al., 2011; Zuloaga et al., 2012a,b; Carbone and Handa, 2013; Seney et al., 2013). Despite these findings, a number of confounds (state vs. trait, treatment, age, and recurrence) present challenges to elucidating the contribution of hormonal or genetic sex (Seney et al., 2013) to the co-occurrence of hormonal dysregulation and mood disorders.

## HPA axis implicated in MDD

A central role for the HPA axis in MDD was initially expressed clinically. Depressive symptoms/MDD co-occurred with endogenously elevated cortisol (Sonino et al., 1998) or exogenously administered corticosteroids (Kelly et al., 1980; Ling et al., 1981). Studies demonstrated elevated levels of cortisol in plasma, CSF, and 24-h urine samples, high CSF corticotrophin releasing hormone (CRH) levels, blunted responses to CRH administration, and non-suppression of cortisol secretion on the dexamethasone suppression test in MDD (Carroll et al., 1976, 1981; Jarrett et al., 1983; Nemeroff et al., 1984; Halbreich et al., 1985; Holsboer et al., 1985; Banki et al., 1987; Evans and Nemeroff, 1987; Rubin et al., 1987; Heim et al., 2001; Newport et al., 2003; Raison and Miller, 2003). HPA axis dysregulation was related to age (Nelson et al., 1984a,b; Bremmer et al., 2007), depression subtype (Brouwer et al., 2005), recurrence (Poor et al., 2004), and treatment response, albeit inconsistently (Nemeroff et al., 1991; De Bellis et al., 1993; Veith et al., 1993; McKay and Zakzanis, 2010). One potential confound was whether HPA axis dysregulation reflected clinical state or diagnostic trait. A meta-analysis of >1500 individuals (Vreeburg et al., 2009), demonstrated that hypercortisolemia, present in currently depressed individuals (Trestman et al., 1993; Ahrens et al., 2008), persisted after recovery (Vreeburg et al., 2009), while other studies reported abnormal blunted cortisol response to stress in recurrent cases (Ahrens et al., 2008). In either case, findings suggested HPA dysregulation as a trait. In contrast, some studies showed resolution of hypercortisolemia with treatment (Vythilingam et al., 2004; Lok et al., 2012), arguing that HPA dysregulation was due to clinical state. Elevated baseline cortisol, enhanced CRH sensitivity, and lack of responsivity to dexamethasone suppression also predicted relapse vulnerability and sustained remission (Zobel et al., 1999; Appelhof et al., 2006; Ising et al., 2007). Despite this evidence, HPA-axis targeted treatments are not reliably effective in MDD, although show some success as anti-depressant adjuncts (Jahn et al., 2004) or improvement of cognitive deficits (Young et al., 2004).

Despite substantial data supporting sex differences in HPA functioning during stress in healthy populations (Kudielka and Kirschbaum, 2005; Goldstein et al., 2010) and MDD women (Holsen et al., 2011, 2013), reports of sex differences in the HPA axis and MDD are inconsistent. Men, but not women, with MDD demonstrated increased ACTH pulsatility (Young et al., 2007a) and elevated cortisol compared with non-depressed men and women (Bremmer et al., 2007; Hinkelmann et al., 2012). However, depressed women vs. men (Poor et al., 2004) and non-depressed women (Young and Altemus, 2004; Chopra et al., 2009) also expressed hypercortisolemia. Study inconsistencies may be related to timing of cortisol assessments or may reflect methodological confounds, such as age of study subjects (e.g., post-menopausal women differ from premenopausal women and thus sex differences differ), chronicity of illness (e.g., sustained illness may produce blunted cortisol response rather than hypercortisolemia), or low statistical power to detect sex differences which may vary in effect size, depending on characteristics of the sample (details next paragraph). Further, genetic background likely affects HPA axis dysregulation, as demonstrated in studies showing increased ACTH and cortisol in males (but not females) homozygotic for the *alpha*(2)-adrenoreceptor gene and females (but not males) homozygotic for the *beta*(2)-adrenoreceptor gene (Haefner et al., 2008). Collectively, these findings offer initial evidence of sex differences in the role of HPA axis in MDD pathophysiology and emphasize the importance of considering genetic variation in HPA axis-associated genes.

Some studies report no effect of sex on HPA axis deficits in MDD (Carroll et al., 1976; Nelson et al., 1984b; Dahl et al., 1989; Maes et al., 1994; Deuschle et al., 1998; Brouwer et al., 2005; Vreeburg et al., 2009), although some of these studies were not designed initially to investigate sex differences, introducing potential confounds, such as: oversampling women (thus small samples of men) and low statistical power to test for sex differences (Brouwer et al., 2005; Young et al., 2007a; Vreeburg et al., 2009; Hinkelmann et al., 2012); lack of control for use of oral contraceptives or estrogen-replacement therapy (Brouwer et al., 2005) affecting plasma cortisol levels (Kirschbaum et al., 1999); and disregard for menstrual cycle phase or menopausal status during data collection. These confounds present significant challenges to understanding study inconsistencies on sex differences in HPA-MDD associations and their implications for women's mental health.

## HPG axis implicated in MDD

Post-puberty adolescence is a key period during which sex differences in MDD begin to emerge, initially during ages 13–15, with the largest increase in late adolescence (e.g., Hankin et al., 1998). However, few studies have focused on understanding why the higher rate of MDD in girls than boys is initiated during this period. This is unfortunate since puberty is an important critical period for brain plasticity likely arising from differential flooding of the brain with gonadal hormones (Schulz et al., 2009), and further sexual differentiation of the brain as the prefrontal cortex fully develops during ages 18–22 years. Evidence for HPG axis-MDD associations also came from studies of polycystic ovarian syndrome (Himelein and Thatcher, 2006) and literature relating women's reproductive biology to mood fluctuations and depression (Steiner, 1992; Bloch et al., 2000; Payne, 2003; Angold and Costello, 2006; Young et al., 2007b; Graziottin and Serafini, 2009; Brummelte and Galea, 2010). Although there has been less examination of HPG deficits in MDD in men, lower testosterone has been reported (Schweiger et al., 1999; Seidman et al., 2001). HPG dysregulation in MDD has included androgens (Baischer et al., 1995; Rubinow and Schmidt, 1996; Schweiger et al., 1999; Seidman et al., 2001; Weiner et al., 2004), estrogens (Young et al., 2000), and pituitary function (Daly et al., 2003). Women with persistent MDD had two times the risk of earlier perimenopausal transition, higher FSH, and lower estradiol levels, suggesting an early decline in ovarian function (Young et al., 2000; Harlow et al., 2003). Further, depressive symptom severity was associated with low estradiol levels (Baischer et al., 1995).

## HPA-HPG interactions implicated in MDD

Dysregulation of HPA and HPG axes interact in MDD. Low levels of estradiol with unopposed progesterone in premenopausal MDD was associated with decreased inhibitory feedback on HPA function during stress, resulting in elevated cortisol in MDD compared to healthy women or men (Young and Altemus, 2004). Transient dysregulation of HPA axis during the luteal menstrual phase was reported in premenstrual syndrome (Rabin et al., 1990; Roca et al., 2003). Further, using functional MRI, our group showed *hypo*activity in stress-responsive regions in *premenopausal* MDD women was significantly associated with decreased estradiol and increased progesterone levels during the late follicular menstrual phase (Holsen et al., 2011). In *perimenopausal* MDD women, these brain regions were associated with *hyper*cortisolemia and *hyper*activity (Holsen et al., 2013). These imaging studies suggest a complex interplay between HPA and HPG axes, dependent on age and cycle timing. From a brain circuitry point of view, MDD involves hypothalamic (HYPO) nuclei (paraventricular and ventromedial), central amygdala (AMYG), hippocampus (HIPP), anterior cingulate, medial and orbital prefrontal cortices (ACC, mPFC, OFC) (Dougherty and Rauch, 1997; Mayberg, 1997; Drevets et al., 2002; Sheline et al., 2002; Rauch et al., 2003), regions dense in glucocorticoid and sex steroid hormone receptors (MacLusky et al., 1987; Clark et al., 1988; Handa et al., 1994; Kawata, 1995; Tobet and Hanna, 1997; Donahue et al., 2000; Östlund et al., 2003). These regions develop in sex-dependent ways, in part driven by gonadal hormones. There is now a substantial body of functional imaging work relating regulation of mood with endocrine function, e.g., Goldstein et al., 2005, 2010; Protopopescu et al., 2005; Amin et al., 2006; Stark et al., 2006; Dreher et al., 2007; Wang et al., 2007; Pruessner et al., 2008; van Wingen et al., 2008a,b, 2009; Root et al., 2009; Andreano and Cahill, 2010.

## Shared mood and endocrine circuitry are sexually dimorphic

*In vivo* imaging and postmortem studies demonstrated sex differences in brain volumes (or nuclei) of regions associated with MDD, although there is little work focused on sexual dimorphisms in MDD *per se*. *In healthy women compared with men*, relative to cerebrum size, findings supported greater relative volumes of HIPP (Filipek et al., 1994; Giedd et al., 1996; Murphy et al., 1996; Goldstein et al., 2001), ACC (Paus et al., 1996; Goldstein et al., 2001), and OFC (Goldstein et al., 2001). *In men*, there are relatively greater volumes of AMYG (Giedd et al., 1996; Goldstein et al., 2001), HYPO (Swaab and Fliers, 1985; Allen et al., 1989; Goldstein et al., 2001), and paracingulate gyrus (Goldstein et al., 2001; Paus et al., 1996). Recently, a number of new studies have emerged further characterizing sex-dependent circuitry (Ruigrok et al., 2013), connectivity (Ingalhalikar et al., 2014), and potential mechanisms (Raznahan et al., 2010; Kang et al., 2011; Goldstein et al., 2013; Lenz et al., 2013; Nguyen et al., 2013). Developmental pathways involve, in part, gonadal hormone regulation, seen in model animal (McEwen, 1983; Simerly et al., 1990; Tobet et al., 1993, 2009; O'Keefe et al., 1995; Park et al., 1996; Tobet and Hanna, 1997; Gorski, 2000; Chung et al., 2006) and human (Goldstein et al., 2001; Raznahan et al., 2010) development. In fact, preclinical studies demonstrated lasting effects of prenatal adverse events on HPA axis and noradrenergic stress systems (Takahashi et al., 1992; Weinstock et al., 1992; Vallee et al., 1997; Weinstock, 1997). These included hypothalamic and hippocampal structure and function (Takahashi et al., 1992; Matsumoto and Arai, 1997; Weinstock, 1997), with effects that occurred through programming a “hyperactive” system more vulnerable to adult depressive and anxiety-like behaviors and autonomic nervous system deficits, among others (Weinstock et al., 1992; Henry et al., 1994; Barker, 1995; Seckl, 2001; Majdic and Tobet, 2011; Zuloaga et al., 2011; Carbone et al., 2012). Analogous to timing of these events in animals, mid-to-late gestation in humans is a particularly vulnerable time for the impact of prenatal events on *sex-dependent* brain development (Tobet et al., 2009; Majdic and Tobet, 2011; Zuloaga et al., 2011; Carbone et al., 2012), and recent preclinical and clinical studies implicated earlier gestation (Mueller and Bale, 2008; Howerton et al., 2013).

Preclinical studies also demonstrated sex differences (greater in females than males) in a number of domains, including: (1) greater placental glucocorticoid transfer (Montano et al., 1993; Fameli et al., 1994); (2) greater immobility in tasks associated with MDD phenotypic behavior (Alonso et al., 2000); (3) increased ACTH, corticosterone, and glucocorticoid receptor binding (Weinstock et al., 1992; McCormick et al., 1995; Regan et al., 2004); (4) increased corticosterone sensitivity (Rhodes and Rubin, 1999); (5) greater susceptibility to changes following loss of GABA_B_ receptor function (McClellan et al., 2010; Stratton et al., 2011); (6) greater susceptibility to cell death in AMYG following developmental exposure to dexamethasone (Zuloaga et al., 2011); and (8) greater susceptibility to diet-induced hepatosteatosis and insulin growth factor-1 deficits (Carbone et al., 2012). In humans, at the level of the brain, there have been fewer studies of sex differences in MDD, although some reported decreased HIPP and increased AMYG volumes, greater in females than males (Vakili et al., 2000; Janssen et al., 2004; Weniger et al., 2006). Collectively, preclinical studies support the hypothesis that prenatal exposures (particularly those implicating stress circuitry pathways) facilitate altered programming of stress-related endocrine and neural circuits implicated in the sex-dependent development of depressive-like behavior. Although parallel studies in humans are still in their infancy, we and others are currently testing the hypothesis that *prenatal maternal* disruption of stress-immune pathways will, in the context of genetic background, result in vulnerability for the sex-dependent risk for MDD in the offspring (Handa et al., 1994; Majdic and Tobet, 2011).

## Conclusions

The number one cause of disability worldwide is MDD, and women are two times the risk of men. This represents ~350 million people worldwide, approximately 16 million in the U.S. alone (WHO October 2012 Fact Sheet). Depression is comorbid with many chronic diseases that are also associated sex differences in risk (Goldstein et al., 2011, 2013). Thus, depression is a major public health problem with substantial economic, social and disease burden that, we argue, requires a sex-dependent lens to understand its pathophysiology. There are key naturalistic opportunities for the study of this higher risk in women, and that is when the brain is differentially flooded with or depleted of gonadal hormones, i.e., fetal development, puberty, pregnancy, and perimenopausal-menopause transition. The evidence briefly discussed here supports the hypothesis that the etiology of sex differences in MDD begins in fetal development and emerges post-puberty. Its onset can be catalyzed by pregnancy (postpartum depression) and the menopausal transition (when there is an increase in MDD onset). The fact that these particular periods during the lifespan have significant implications for MDD onset is consistent with an important role for steroid hormones in MDD. This underscores the importance of promoting further inquiry into the development of adjunctive neuroendocrine treatments, dependent on timing across the lifespan. This lifespan approach to studying sex differences in disorders, like depression, also illustrates how maternal health (e.g., pregnancy), women's mental health, and sex differences in disorders of the brain are linked. Thus, we have argued the importance for preclinical and clinical neuroscience to incorporate a sex-dependent and/or sex-specific lens on investigations ranging from the cellular-molecular level to circuitry, systems, and behavior, an argument that recently was underscored by the new directive from NIH to incorporate this perspective in designs of preclinical studies (Clayton and Collins, 2014). We believe this will provide the basis for the development of sex-dependent therapeutics which will enhance progress to greater efficacy.

### Conflict of interest statement

The authors declare that the research was conducted in the absence of any commercial or financial relationships that could be construed as a potential conflict of interest.

## References

[B1] Ahrens T. Deuschle M. Krumm B. van der Pompe G. den Boer J. A. Lederbogen F. (2008). Pituitary-adrenal and sympathetic nervous system responses to stress in women remitted from recurrent major depression. Psychosom. Med. 70, 461–467 10.1097/PSY.0b013e31816b1aaa 18378864

[B2] Allen L. S. Hines M. Shryne J. E. Gorski R. A. (1989). Two sexually dimorphic cell groups in the human brain. J. Neurosci. 9, 497–506 2918374 10.1523/JNEUROSCI.09-02-00497.1989 PMC6569815

[B3] Alonso S. J. Damas C. Navarro E. (2000). Behavioral despair in mice after prenatal stress. J. Physiol. Biochem. 56, 77–82 10.1007/BF03179902 11014612

[B4] Amin Z. Epperson C. N. Constable R. T. Canli T. (2006). Effects of estrogen variation on neural correlates of emotional response inhibition. Neuroimage 32, 457–464 10.1016/j.neuroimage.2006.03.013 16644236

[B5] Andreano J. M. Cahill L. (2010). Menstrual cycle modulation of medial temporal activity evoked by negative emotion. Neuroimage 53, 1286–1293 10.1016/j.neuroimage.2010.07.011 20637290 PMC3376005

[B6] Angold A. Costello E. J. (2006). Puberty and depression. Child Adolesc. Psychiatr. Clin. N. Am. 15, 919–937, ix. 10.1016/j.chc.2006.05.013 16952768

[B7] Appelhof B. C. Huyser J. Verweij M. Brouwer J. P. van Dyck R. Fliers E. (2006). Glucocorticoids and relapse of major depression (dexamethasone/corticotropin-releasing hormone test in relation to relapse of major depression). Biol. Psychiatry 59, 696–701 10.1016/j.biopsych.2005.09.008 16368077

[B8] Baischer W. Koinig G. Hartmann B. Huber J. Langer G. (1995). Hypothalamic-pituitary-gonadal axis in depressed premenopausal women: elevated blood testosterone concentrations compared to normal controls. Psychoneuroendocrinology 20, 553–559 10.1016/0306-4530(94)00081-K 7675939

[B9] Banki C. M. Bissette G. Arato M. O'Connor L. Nemeroff C. B. (1987). CSF corticotropin-releasing factor-like immunoreactivity in depression and schizophrenia. Am. J. Psychiatry 144, 873–877 3496802 10.1176/ajp.144.7.873

[B10] Barim A. O. Aydin S. Colak R. Dag E. Deniz O. Sahin I. (2009). Ghrelin, paraoxonase and arylesterase levels in depressive patients before and after citalopram treatment. Clin. Biochem. 42, 1076–1081 10.1016/j.clinbiochem.2009.02.020 19272368

[B11] Barker D. J. (1995). Intrauterine programming of adult disease. Mol. Med. Today 1, 418–423 10.1016/S1357-4310(95)90793-9 9415190

[B12] Bloch M. Schmidt P. J. Danaceau M. Murphy J. Nieman L. Rubinow D. R. (2000). Effects of gonadal steroids in women with a history of postpartum depression. Am. J. Psychiatry 157, 924–930 10.1176/appi.ajp.157.6.924 10831472

[B13] Board F. Persky H. Hamburg D. A. (1956). Psychological stress and endocrine functions; blood levels of adrenocortical and thyroid hormones in acutely disturbed patients. Psychosom. Med. 18, 324–333 10.1097/00006842-195607000-00006 13350458

[B14] Bremmer M. A. Deeg D. J. Beekman A. T. Penninx B. W. Lips P. Hoogendijk W. J. (2007). Major depression in late life is associated with both hypo- and hypercortisolemia. Biol. Psychiatry 62, 479–886 10.1016/j.biopsych.2006.11.033 17481591

[B15] Brouwer J. P. Appelhof B. C. Hoogendijk W. J. Huyser J. Endert E. Zuketto C. (2005). Thyroid and adrenal axis in major depression: a controlled study in outpatients. Eur. J. Endocrinol. 152, 185–191 10.1530/eje.1.01828 15745924

[B16] Brummelte S. Galea L. A. (2010). Depression during pregnancy and postpartum: contribution of stress and ovarian hormones. Prog. Neuropsychopharmacol. Biol. Psychiatry 34, 766–776 10.1016/j.pnpbp.2009.09.006 19751794

[B17] Carbone D. L. Handa R. J. (2013). Sex and stress hormone influences on the expression and activity of brain-derived neurotrophic factor. Neuroscience 239, 295–303 10.1016/j.neuroscience.2012.10.073 23211562 PMC3609934

[B18] Carbone D. L. Zuloaga D. G. Hiroi R. Foradori C. D. Legare M. E. Handa R. J. (2012). Prenatal dexamethasone exposure potentiates diet-induced hepatosteatosis and decreases plasma IGF-I in a sex-specific fashion. Endocrinology 153, 295–306 10.1210/en.2011-1601 22067322 PMC3249671

[B19] Carroll B. J. Curtis G. C. Mendels J. (1976). Cerebrospinal fluid and plasma free cortisol concentrations in depression. Psychol. Med. 6, 235–244 10.1017/S0033291700013775 1005564

[B20] Carroll B. J. Feinberg M. Greden J. F. Tarika J. Albala A. A. Haskett R. F. (1981). A specific laboratory test for the diagnosis of melancholia. standardization, validation, and clinical utility. Arch. Gen. Psychiatry 38, 15–22 10.1001/archpsyc.1981.01780260017001 7458567

[B21] Chopra K. K. Ravindran A. Kennedy S. H. Mackenzie B. Matthews S. Anisman H. (2009). Sex differences in hormonal responses to a social stressor in chronic major depression. Psychoneuroendocrinology 34, 1235–1241 10.1016/j.psyneuen.2009.03.014 19386421

[B22] Chung W. C. Pak T. R. Weiser M. J. Hinds L. R. Andersen M. E. Handa R. J. (2006). Progestin receptor expression in the developing rat brain depends upon activation of estrogen receptor alpha and not estrogen receptor beta. Brain Res. Mol. Brain Res. 1082, 50–60 10.1016/j.brainres.2006.01.109 16513095

[B23] Clark A. S. MacLusky N. J. Goldman-Rakic P. S. (1988). Androgen binding and metabolism in the cerebral cortex of the developing rhesus monkey. Endocrinology 123, 932–940 10.1210/endo-123-2-932 3260856

[B24] Clayton J. A. Collins F. S. (2014). NIH to balance sex in cell and animal studies. Nature 509, 282–283 10.1038/509282a 24834516 PMC5101948

[B25] Coplan J. D. Wolk S. I. Goetz R. R. Ryan N. D. Dahl R. E. Mann J. J. (2000). Nocturnal growth hormone secretion studies in adolescents with or without major depression re-examined: integration of adult clinical follow-up data. Biol. Psychiatry 47, 594–604 10.1016/S0006-3223(00)00226-2 10745051

[B26] Dahl R. Puig-Antich J. Ryan N. Nelson B. Novacenko H. Twomey J. (1989). Cortisol secretion in adolescents with major depressive disorder. Acta Psychiatr. Scand. 80, 18–26 10.1111/j.1600-0447.1989.tb01295.x 2763857

[B27] Daly R. C. Danaceau M. A. Rubinow D. R. Schmidt P. J. (2003). Concordant restoration of ovarian function and mood in perimenopausal depression. Am. J. Psychiatry 160, 1842–1846 10.1176/appi.ajp.160.10.1842 14514500

[B28] De Bellis M. D. Gold P. W. Geracioti T. D. Jr. Listwak S. J. Kling M. A. (1993). Association of fluoxetine treatment with reductions in CSF concentrations of corticotropin-releasing hormone and arginine vasopressin in patients with major depression. Am. J. Psychiatry 150, 656–657 8465888 10.1176/ajp.150.4.656

[B29] Deuschle M. Weber B. Colla M. Depner M. Heuser I. (1998). Effects of major depression, aging and gender upon calculated diurnal free plasma cortisol concentrations: a re-evaluation study. Stress 2, 281–287 10.3109/10253899809167292 9876259

[B30] Donahue J. E. Stopa E. G. Chorsky R. L. King J. C. Schipper H. M. Tobet S. A. (2000). Cells containing immunoreactive estrogen receptor-alpha in the human basal forebrain. Brain Res. Mol. Brain Res. 856, 142–151 10.1016/S0006-8993(99)02413-0 10677621

[B31] Dougherty D. Rauch S. L. (1997). Neuroimaging and neurobiological models of depression. Harv. Rev. Psychiatry 5, 138–159 10.3109/10673229709000299 9385033

[B32] Dreher J. C. Schmidt P. J. Kohn P. Furman D. Rubinow D. Berman K. F. (2007). Menstrual cycle phase modulates reward-related neural function in women. Proc. Natl. Acad. Sci. U.S.A. 104, 2465–2470 10.1073/pnas.0605569104 17267613 PMC1892961

[B33] Drevets W. C. Price J. L. Bardgett M. E. Reich T. Todd R. D. Raichle M. E. (2002). Glucose metabolism in the amygdala in depression: relationship to diagnostic subtype and plasma cortisol levels. Pharmacol. Biochem. Behav. 71, 431–447 10.1016/S0091-3057(01)00687-6 11830178

[B34] Evans D. L. Nemeroff C. B. (1987). The clinical use of the dexamethasone suppression test in DSM-III affective disorders: correlation with the severe depressive subtypes of melancholia and psychosis. J. Psychiatr. Res. 21, 185–194 10.1016/0022-3956(87)90018-5 3585807

[B35] Fameli M. Kitraki E. Stylianopoulou F. (1994). Effects of hyperactivity of the maternal hypothalamic-pituitary-adrenal (HPA) axis during pregnancy on the development of the HPA axis and brain monoamines of the offspring. Int. J. Dev. Neurosci. 12, 651–659 10.1016/0736-5748(94)90017-5 7900547

[B36] Filipek P. A. Richelme C. Kennedy D. N. Caviness V. S. Jr. (1994). The young adult human brain: an MRI-based morphometric analysis. Cereb. Cortex 4, 344–360 10.1093/cercor/4.4.344 7950308

[B37] Gibbons J. L. McHugh P. R. (1962). Plasma cortisol in depressive illness. J. Psychiatr. Res. 1, 162–171 10.1016/0022-3956(62)90006-7 13947658

[B38] Giedd J. N. Vaituzis A. C. Hamburger S. D. Lange N. Rajapakse J. C. Kaysen D. (1996). Quantitative MRI of the temporal lobe, amygdala, and hippocampus in normal human development: ages 4-18 years. J. Comp. Neurol. 366, 223–230 8698883 10.1002/(SICI)1096-9861(19960304)366:2<223::AID-CNE3>3.0.CO;2-7

[B39] Goldstein J. M. Cherkerzian S. Buka S. L. Fitzmaurice G. Hornig M. Gillman M. (2011). Sex-specific impact of maternal-fetal risk factors on depression and cardiovascular risk 40 years later. J. Dev. Orig. Health Dis. 2, 353–364 10.1017/S2040174411000651 23378891 PMC3558934

[B41] Goldstein J. M. Handa R. J. Tobet S. A. (2013). Disruption of fetal hormonal programming (prenatal stress) implicates shared risk for sex differences in depression and cardiovascular disease. Front. Neuroendocrinol. 35:140–158 10.1016/j.yfrne.2013.12.001 24355523 PMC3917309

[B42] Goldstein J. M. Jerram M. Abbs B. Whitfield-Gabrieli S. Makris N. (2010). Sex differences in stress response circuitry activation dependent on female hormonal cycle. J. Neurosci. 30, 431–438 10.1523/JNEUROSCI.3021-09.2010 20071507 PMC2827936

[B43] Goldstein J. M. Jerram M. Poldrack R. Ahern T. Kennedy D. N. Seidman L. J. (2005). Hormonal cycle modulates arousal circuitry in women using functional magnetic resonance imaging. J. Neurosci. 25, 9309–9316 10.1523/JNEUROSCI.2239-05.2005 16207891 PMC6725775

[B44] Goldstein J. M. Seidman L. J. Horton N. J. Makris N. Kennedy D. N. Caviness V. S. Jr. (2001). Normal sexual dimorphism of the adult human brain assessed by *in vivo* magnetic resonance imaging. Cereb. Cortex 11, 490–497 10.1093/cercor/11.6.490 11375910

[B45] Gorski R. A. (2000). Sexual differentiation of the nervous system, in Principles of Neural Science, eds Kandel E. R. Schwartz J. H. Jessell T. M (New York, NY: McGraw-Hill Health Professions Division), 1131–1146

[B46] Graziottin A. Serafini A. (2009). Depression and the menopause: why antidepressants are not enough? Menopause Int. 15, 76–81 10.1258/mi.2009.009021 19465674

[B47] Haefner S. Baghai T. C. Schule C. Eser D. Spraul M. Zill P. (2008). Impact of gene-gender effects of adrenergic polymorphisms on hypothalamic-pituitary-adrenal axis activity in depressed patients. Neuropsychobiology 58, 154–162 10.1159/000182891 19088492

[B48] Halbreich U. Asnis G. M. Shindledecker R. Zumoff B. Nathan R. S. (1985). Cortisol secretion in endogenous depression. I. Basal plasma levels. Arch. Gen. Psychiatry 42, 904–908 10.1001/archpsyc.1985.01790320076010 4037990

[B49] Handa R. J. Burgess L. H. Kerr J. E. O'Keefe J. A. (1994). Gonadal steroid hormone receptors and sex differences in the hypothalamo-pituitary-adrenal axis. Horm. Behav. 28, 464–476 10.1006/hbeh.1994.1044 7729815

[B50] Hankin B. L. Abramson L. Y. Moffitt T. E. Silva P. A. McGee R. Angell K. E. (1998). Development of depression from preadolescence to young adulthood: emerging gender differences in a 10-year longitudinal study. J. Abnorm. Psychol. 107, 128 10.1037/0021-843X.107.1.128 9505045

[B51] Harlow B. L. Wise L. A. Otto M. W. Soares C. N. Cohen L. S. (2003). Depression and its influence on reproductive endocrine and menstrual cycle markers associated with perimenopause: the harvard study of moods and cycles. Arch. Gen. Psychiatry 60, 29–36 10.1001/archpsyc.60.1.29 12511170

[B52] Heim C. Newport D. J. Bonsall R. Miller A. H. Nemeroff C. B. (2001). Altered pituitary-adrenal axis responses to provocative challenge tests in adult survivors of childhood abuse. Am. J. Psychiatry 158, 575–581 10.1176/appi.ajp.158.4.575 11282691

[B53] Henry C. Kabbaj M. Simon H. Le Moal M. Maccari S. (1994). Prenatal stress increases the hypothalamo-pituitary-adrenal axis response in young and adult rats. J. Neuroendocrinol. 6, 341–345 10.1111/j.1365-2826.1994.tb00591.x 7920600

[B54] Himelein M. J. Thatcher S. S. (2006). Polycystic ovary syndrome and mental health: a review. Obstet. Gynecol. Surv. 61, 723–732 10.1097/01.ogx.0000243772.33357.84 17044949

[B55] Hinkelmann K. Botzenhardt J. Muhtz C. Agorastos A. Wiedemann K. Kellner M. (2012). Sex differences of salivary cortisol secretion in patients with major depression. Stress 15, 105–109 10.3109/10253890.2011.582200 21790344

[B56] Holsboer F. Gerken A. Stalla G. K. Muller O. A. (1985). ACTH, cortisol, and corticosterone output after ovine corticotropin-releasing factor challenge during depression and after recovery. Biol. Psychiatry 20, 276–286 10.1016/0006-3223(85)90057-5 2983788

[B57] Holsen L. M. Lancaster K. Klibanski A. Whitfield-Gabrieli S. Cherkerzian S. Buka S. (2013). HPA-axis hormone modulation of stress response circuitry activity in women with remitted major depression. Neuroscience 250, 733–742 10.1016/j.neuroscience.2013.07.042 23891965 PMC3772711

[B58] Holsen L. M. Spaeth S. B. Lee J. H. Ogden L. A. Klibanski A. Whitfield-Gabrieli S. (2011). Stress response circuitry hypoactivation related to hormonal dysfunction in women with major depression. J. Affect. Disord. 131, 379–387 10.1016/j.jad.2010.11.024 21183223 PMC3073153

[B59] Howerton C. L. Morgan C. P. Fischer D. B. Bale T. L. (2013). O-GlcNAc transferase (OGT) as a placental biomarker of maternal stress and reprogramming of CNS gene transcription in development. Proc. Natl. Acad. Sci.U.S.A. 110, 5169–5174 10.1073/pnas.1300065110 23487789 PMC3612602

[B60] Ingalhalikar M. Smith A. Parker D. Satterthwaite T. D. Elliott M. A. Ruparel K. (2014). Sex differences in the structural connectome of the human brain. Proc. Natl. Acad. Sci.U.S.A. 111, 823–828 10.1073/pnas.1316909110 24297904 PMC3896179

[B61] Ising M. Horstmann S. Kloiber S. Lucae S. Binder E. B. Kern N. (2007). Combined dexamethasone/corticotropin releasing hormone test predicts treatment response in major depression - a potential biomarker? Biol. Psychiatry 62, 47–54 10.1016/j.biopsych.2006.07.039 17123470

[B62] Jahn H. Schick M. Kiefer F. Kellner M. Yassouridis A. Wiedemann K. (2004). Metyrapone as additive treatment in major depression: a double-blind and placebo-controlled trial. Arch. Gen. Psychiatry 61, 1235–1244 10.1001/archpsyc.61.12.1235 15583115

[B63] Janssen J. Hulshoff Pol H. E. Lampe I. K. Schnack H. G. de Leeuw F. E. Kahn R. S. (2004). Hippocampal changes and white matter lesions in early-onset depression. Biol. Psychiatry 56, 825–831 10.1016/j.biopsych.2004.09.011 15576058

[B64] Jarrett D. B. Coble P. A. Kupfer D. J. (1983). Reduced cortisol latency in depressive illness. Arch. Gen. Psychiatry 40, 506–511 10.1001/archpsyc.1983.01790050032004 6838331

[B65] Kang H. J. Kawasawa Y. I. Cheng F. Zhu Y. Xu X. Li M. (2011). Spatio-temporal transcriptome of the human brain. Nature 478, 483–489 10.1038/nature10523 22031440 PMC3566780

[B66] Kawata M. (1995). Roles of steroid hormones and their receptors in structural organization in the nervous system. Neurosci. Res. 24, 1–46 10.1016/0168-0102(96)81278-8 8848287

[B67] Kelly W. F. Checkley S. A. Bender D. A. (1980). Cushing's syndrome, tryptophan and depression. Br. J. Psychiatry 136, 125–132 10.1192/bjp.136.2.125 7370478

[B68] Kendler K. S. Gatz M. Gardner C. O. Pedersen N. L. (2006). A Swedish national twin study of lifetime major depression. Am. J. Psychiatry 163, 109–114 10.1176/appi.ajp.163.1.109 16390897

[B69] Kessler R. C. (2003). Epidemiology of women and depression. J. Affect. Disord. 74, 5–13 10.1016/S0165-0327(02)00426-3 12646294

[B70] Kirschbaum C. Kudielka B. M. Gaab J. Schommer N. C. Hellhammer D. H. (1999). Impact of gender, menstrual cycle phase, and oral contraceptives on the activity of the hypothalamus-pituitary-adrenal axis. Psychosom. Med. 61, 154–162 10.1097/00006842-199903000-00006 10204967

[B71] Kudielka B. M. Kirschbaum C. (2005). Sex differences in HPA axis responses to stress: a review. Biol. Psychol. 69, 113–132 10.1016/j.biopsycho.2004.11.009 15740829

[B72] Kurt E. Guler O. Serteser M. Cansel N. Ozbulut O. Altinbas K. (2007). The effects of electroconvulsive therapy on ghrelin, leptin and cholesterol levels in patients with mood disorders. Neurosci. Lett. 426, 49–53 10.1016/j.neulet.2007.08.018 17884293

[B73] Lenz K. M. Nugent B. M. Haliyur R. McCarthy M. M. (2013). Microglia are essential to masculinization of brain and behavior. J. Neurosci. 33, 2761–2772 10.1523/JNEUROSCI.1268-12.2013 23407936 PMC3727162

[B74] Ling M. H. Perry P. J. Tsuang M. T. (1981). Side effects of corticosteroid therapy. Psychiatric aspects. Arch. Gen. Psychiatry 38, 471–417 10.1001/archpsyc.1981.01780290105011 7212976

[B75] Lok A. Mocking R. J. Ruhe H. G. Visser I. Koeter M. W. Assies J. (2012). Longitudinal hypothalamic-pituitary-adrenal axis trait and state effects in recurrent depression. Psychoneuroendocrinology 37, 892–902 10.1016/j.psyneuen.2011.10.005 22094110

[B76] MacLusky N. J. Clark A. S. Naftolin F. Goldman-Rakic P. S. (1987). Estrogen formation in the mammalian brain: possible role of aromatase in sexual differentiation of the hippocampus and neocortex. Steroids 50, 459–474 10.1016/0039-128X(87)90032-8 3332936

[B77] Maes M. Calabrese J. Meltzer H. Y. (1994). The relevance of the in- versus outpatient status for studies on HPA-axis in depression: spontaneous hypercortisolism is a feature of major depressed inpatients and not of major depression *per se*. Prog. Neuropsychopharmacol. Biol. Psychiatry 18, 503–517 10.1016/0278-5846(94)90008-6 8078985

[B78] Majdic G. Tobet S. (2011). Cooperation of sex chromosomal genes and endocrine influences for hypothalamic sexual differentiation. Front. Neuroendocrinol. 32:137–145 10.1016/j.yfrne.2011.02.009 21338619 PMC3085655

[B79] Matsumoto A. Arai Y. (1997). Sexual differentiation of neuronal circuitry in the neuroendocrine hypothalamus. Biomed. Rev. 7, 5–15 10.14748/bmr.v7.158

[B80] Mayberg H. S. (1997). Limbic-cortical dysregulation: a proposed model of depression. J. Neuropsychiatry Clin. Neurosci. 9, 471–481 9276848 10.1176/jnp.9.3.471

[B81] McClellan K. M. Stratton M. S. Tobet S. A. (2010). Roles for gamma-aminobutyric acid in the development of the paraventricular nucleus of the hypothalamus. J. Comp. Neurol. 518, 2710–2728 10.1002/cne.22360 20506472 PMC2879086

[B82] McCormick C. M. Smythe J. W. Sharma S. Meaney M. J. (1995). Sex-specific effects of prenatal stress on hypothalamic-pituitary-adrenal responses to stress and brain glucocorticoid receptor density in adult rats. Brain Res. Dev. Brain Res. 84, 55–61 10.1016/0165-3806(94)00153-Q 7720217

[B83] McEwen B. S. (1983). Gonadal steroid influences on brain development and sexual differentiation, in Reproductive Physiology IV, ed Greep R. (University Park: Baltimore), 99–145 6303978

[B84] McKay M. S. Zakzanis K. K. (2010). The impact of treatment on HPA axis activity in unipolar major depression. J. Psychiatr. Res. 44, 183–192 10.1016/j.jpsychires.2009.07.012 19747693

[B85] Montano M. M. Wang M. H. vom Saal F. S. (1993). Sex differences in plasma corticosterone in mouse fetuses are mediated by differential placental transport from the mother and eliminated by maternal adrenalectomy or stress. J. Reprod. Fertil. 99, 283–290 10.1530/jrf.0.0990283 8107008

[B86] Mueller B. R. Bale T. L. (2008). Sex-specific programming of offspring emotionality after stress early in pregnancy. J. Neurosci. 28, 9055–9065 10.1523/JNEUROSCI.1424-08.2008 18768700 PMC2731562

[B87] Murphy D. G. DeCarli C. McIntosh A. R. Daly E. Mentis M. J. Pietrini P. (1996). Sex differences in human brain morphometry and metabolism: an *in vivo* quantitative magnetic resonance imaging and positron emission tomography study on the effect of aging. Arch. Gen. Psychiatry 53, 585–594 10.1001/archpsyc.1996.01830070031007 8660125

[B88] Murray C. J. Lopez A. D. (1997). Mortality by cause for eight regions of the world: global burden of disease study. Lancet 349, 1269–1276 10.1016/S0140-6736(96)07493-4 9142060

[B89] Nelson W. H. Khan A. Orr W. W. Jr. Tamragouri R. N. (1984a). The dexamethasone suppression test: interaction of diagnosis, sex, and age in psychiatric inpatients. Biol. Psychiatry 19, 1293–1304 6498252

[B90] Nelson W. H. Orr W. W. Jr. Shane S. R. Stevenson J. M. (1984b). Hypothalamic-pituitary-adrenal axis activity and age in major depression. J. Clin. Psychiatry 45, 120–121 6698943

[B91] Nemeroff C. B. Bissette G. Akil H. Fink M. (1991). Neuropeptide concentrations in the cerebrospinal fluid of depressed patients treated with electroconvulsive therapy. Corticotrophin-releasing factor, beta-endorphin and somatostatin. Br. J. Psychiatry 158, 59–63 10.1192/bjp.158.1.59 1673078

[B92] Nemeroff C. B. Widerlov E. Bissette G. Walleus H. Karlsson I. Eklund K. (1984). Elevated concentrations of CSF corticotropin-releasing factor-like immunoreactivity in depressed patients. Science 226, 1342–1344 10.1126/science.6334362 6334362

[B93] Newport D. J. Heim C. Owens M. J. Ritchie J. C. Ramsey C. H. Bonsall R. (2003). Cerebrospinal fluid corticotropin-releasing factor (CRF) and vasopressin concentrations predict pituitary response in the CRF stimulation test: a multiple regression analysis. Neuropsychopharmacology 28, 569–576 10.1038/sj.npp.1300071 12629539

[B94] Nguyen T. V. McCracken J. Ducharme S. Botteron K. N. Mahabir M. Johnson W. (2013). Testosterone-related cortical maturation across childhood and adolescence. Cereb. Cortex 23, 1424–1432 10.1093/cercor/bhs125 22617851 PMC3643718

[B95] O'Keefe J. A. Li Y. Burgess L. H. Handa R. J. (1995). Estrogen receptor mRNA alterations in the developing rat hippocampus. Brain Res. Mol. Brain Res. 30, 115–124 10.1016/0169-328X(94)00284-L 7609632

[B96] Östlund H. Keller E. Hurd Y. L. (2003). Estrogen receptor gene expression in relation to neuropsychiatric disorders. Ann. N.Y. Acad. Sci. 1007, 54–63 10.1196/annals.1286.006 14993040

[B97] Park J. J. Baum M. J. Paredes R. G. Tobet S. A. (1996). Neurogenesis and cell migration into the sexually dimorphic preoptic area/anterior hypothalamus of the fetal ferret. J. Neurobiol. 30, 315–328 8807525 10.1002/(SICI)1097-4695(199607)30:3<315::AID-NEU1>3.0.CO;2-7

[B98] Paus T. Otaky N. Caramanos Z. MacDonald D. Zijdenbos A. D'Avirro D. (1996). *In vivo* morphometry of the intrasulcal gray matter in the human cingulate, paracingulate, and superior-rostral sulci: hemispheric asymmetries, gender differences and probability maps. J. Comp. Neurol. 376, 664–673 8978477 10.1002/(SICI)1096-9861(19961223)376:4<664::AID-CNE12>3.0.CO;2-M

[B99] Payne J. L. (2003). The role of estrogen in mood disorders in women. Int. Rev. Psychiatry 15, 280–290 10.1080/0954026031000136893 15276966

[B100] Plotsky P. M. Owens M. J. Nemeroff C. B. (1998). Psychoneuroendocrinology of depression. Hypothalamic-pituitary-adrenal axis. Psychiatr. Clin. N. Am. 21, 293–307 10.1016/S0193-953X(05)70006-X 9670227

[B101] Poor V. Juricskay S. Gati A. Osvath P. Tenyi T. (2004). Urinary steroid metabolites and 11beta-hydroxysteroid dehydrogenase activity in patients with unipolar recurrent major depression. J. Affect. Disord. 81, 55–59 10.1016/S0165-0327(03)00199-X 15183600

[B102] Protopopescu X. Pan H. Altemus M. Tuescher O. Polanecsky M. McEwen B. (2005). Orbitofrontal cortex activity related to emotional processing changes across the menstrual cycle. Proc. Natl. Acad. Sci. U.S.A. 102, 16060–16065 10.1073/pnas.0502818102 16247013 PMC1276043

[B103] Pruessner J. C. Dedovic K. Khalili-Mahani N. Engert V. Pruessner M. Buss C. (2008). Deactivation of the limbic system during acute psychosocial stress: evidence from positron emission tomography and functional magnetic resonance imaging studies. Biol. Psychiatry 63, 234–240 10.1016/j.biopsych.2007.04.041 17686466

[B104] Rabin D. S. Schmidt P. J. Campbell G. Gold P. W. Jensvold M. Rubinow D. R. (1990). Hypothalamic-pituitary-adrenal function in patients with the premenstrual syndrome. J. Clin. Endocrinol. Metab. 71, 1158–1162 10.1210/jcem-71-5-1158 2172272

[B105] Raison C. L. Miller A. H. (2003). When not enough is too much: the role of insufficient glucocorticoid signaling in the pathophysiology of stress-related disorders. Am. J. Psychiatry 160, 1554–1565 10.1176/appi.ajp.160.9.1554 12944327

[B106] Rauch S. L. Shin L. M. Wright C. I. (2003). Neuroimaging studies of amygdala function in anxiety disorders. Ann. N.Y. Acad. Sci. 985, 389–410 10.1111/j.1749-6632.2003.tb07096.x 12724173

[B107] Raznahan A. Lee Y. Stidd R. Long R. Greenstein D. Clasen L. (2010). Longitudinally mapping the influence of sex and androgen signaling on the dynamics of human cortical maturation in adolescence. Proc. Natl. Acad. Sci. U.S.A. 107, 16988–16993 10.1073/pnas.1006025107 20841422 PMC2947865

[B108] Regan J. Wagner D. Hamer G. Wright A. White C. (2004). Latinos and their mental health. Tennessee Med. 97, 218–219 15162573

[B109] Rhodes M. E. Rubin R. T. (1999). Functional sex differences (“sexual diergism”) of central nervous system cholinergic systems, vasopressin, and hypothalamic-pituitary-adrenal axis activity in mammals: a selective review. Brain Res. Brain Res. Rev. 30, 135–152 10.1016/S0165-0173(99)00011-9 10525171

[B110] Roca C. A. Schmidt P. J. Altemus M. Deuster P. Danaceau M. A. Putnam K. (2003). Differential menstrual cycle regulation of hypothalamic-pituitary-adrenal axis in women with premenstrual syndrome and controls. J. Clin. Endocrinol. Metab. 88, 3057–3063 10.1210/jc.2002-021570 12843143

[B111] Root J. C. Tuescher O. Cunningham-Bussel A. Pan H. Epstein J. Altemus M. (2009). Frontolimbic function and cortisol reactivity in response to emotional stimuli. Neuroreport 20, 429–434 10.1097/WNR.0b013e328326a031 19225430

[B112] Rubin R. T. Poland R. E. Lesser I. M. Winston R. A. Blodgett A. L. (1987). Neuroendocrine aspects of primary endogenous depression. I. Cortisol secretory dynamics in patients and matched controls. Arch. Gen. Psychiatry 44, 328–336 10.1001/archpsyc.1987.01800160032006 3566455

[B113] Rubinow D. R. Schmidt P. J. (1996). Androgens, brain, and behavior. Am. J. Psychiatry 153, 974–884 8678193 10.1176/ajp.153.8.974

[B114] Ruigrok A. N. Salimi-Khorshidi G. Lai M. C. Baron-Cohen S. Lombardo M. V. Tait R. J. (2013). A meta-analysis of sex differences in human brain structure. Neurosci. Biobehav. Rev. 39, 34–50 10.1016/j.neubiorev.2013.12.004 24374381 PMC3969295

[B115] Schulz K. M. Molenda-Figueira H. A. Sisk C. L. (2009). Back to the future: the organizational-activational hypothesis adapted to puberty and adolescence. Horm. Behav. 55, 597–604 10.1016/j.yhbeh.2009.03.010 19446076 PMC2720102

[B116] Schweiger U. Deuschle M. Weber B. Korner A. Lammers C. H. Schmider J. (1999). Testosterone, gonadotropin, and cortisol secretion in male patients with major depression. Psychosom. Med. 61, 292–296 10.1097/00006842-199905000-00007 10367608

[B117] Seckl J. R. (2001). Glucocorticoid programming of the fetus; adult phenotypes and molecular mechanisms. Mol. Cell. Endocrinol. 185, 61–71 10.1016/S0303-7207(01)00633-5 11738795

[B118] Seidman S. N. Araujo A. B. Roose S. P. McKinlay J. B. (2001). Testosterone level, androgen receptor polymorphism, and depressive symptoms in middle-aged men. Biol. Psychiatry 50, 371–376 10.1016/S0006-3223(01)01148-9 11543741

[B119] Seney M. L. Ekong K. I. Ding Y. Tseng G. C. Sibille E. (2013). Sex chromosome complement regulates expression of mood-related genes. Biol. Sex Differ. 4, 20 10.1186/2042-6410-4-20 24199867 PMC4175487

[B120] Sheline Y. I. Mittler B. L. Mintun M. A. (2002). The hippocampus and depression. Eur. Psychiatry 17 Suppl. 3, 300–305 10.1016/S0924-9338(02)00655-7 15177085

[B121] Simerly R. B. Chang C. Muramatsu M. Swanson L. W. (1990). Distribution of androgen and estrogen receptor mRNA-containing cells in the rat brain: an *in situ* hybridization study. J. Comp. Neurol. 294, 76–95 10.1002/cne.902940107 2324335

[B122] Sonino N. Fava G. A. Raffi A. R. Boscaro M. Fallo F. (1998). Clinical correlates of major depression in Cushing's disease. Psychopathology 31, 302–306 10.1159/000029054 9780396

[B123] Stark R. Wolf O. T. Tabbert K. Kagerer S. Zimmermann M. Kirsch P. (2006). Influence of the stress hormone cortisol on fear conditioning in humans: evidence for sex differences in the response of the prefrontal cortex. Neuroimage 32, 1290–1298 10.1016/j.neuroimage.2006.05.046 16839780

[B124] Steiner M. (1992). Female-specific mood disorders. Clin. Obstet. Gynecol. 35, 599–611 10.1097/00003081-199209000-00020 1521388

[B125] Stratton M. S. Searcy B. T. Tobet S. A. (2011). GABA regulates corticotropin releasing hormone levels in the paraventricular nucleus of the hypothalamus in newborn mice. Physiol. Behav. 104, 327–333 10.1016/j.physbeh.2011.01.003 21236282 PMC3108002

[B126] Swaab D. F. Bao A. M. Lucassen P. J. (2005). The stress system in the human brain in depression and neurodegeneration. Ageing Res. Rev. 4, 141–194 10.1016/j.arr.2005.03.003 15996533

[B127] Swaab D. F. Fliers E. (1985). A sexually dimorphic nucleus in the human brain. Science 228, 1112–1125 10.1126/science.3992248 3992248

[B128] Takahashi A. Sudo M. Minokoshi Y. Shimazu T. (1992). Effects of ventromedial hypothalamic stimulation on glucose transport system in rat tissues. Am. J. Physiol. 263, R1228–R1234 1481931 10.1152/ajpregu.1992.263.6.R1228

[B129] Tobet S. A. Basham M. E. Baum M. J. (1993). Estrogen receptor immunoreactive neurons in the fetal ferret forebrain. Brain Res. Dev. Brain Res. 72, 167–180 10.1016/0165-3806(93)90182-A 8485841

[B130] Tobet S. A. Hanna I. K. (1997). Ontogeny of sex differences in the mammalian hypothalamus and preoptic area. Cell. Mol. Neurobiol. 17, 565–601 10.1023/A:1022529918810 9442348 PMC11560209

[B131] Tobet S. Knoll J. G. Hartshorn C. Aurand E. Stratton M. Kumar P. (2009). Brain sex differences and hormone influences: a moving experience? J. Neuroendocrinol. 21, 387–392 10.1111/j.1365-2826.2009.01834.x 19207813 PMC2669491

[B132] Trestman R. L. Coccaro E. F. Mitropoulou V. Gabriel S. M. Horvath T. Siever L. J. (1993). The cortisol response to clonidine in acute and remitted depressed men. Biol. Psychiatry 34, 373–379 10.1016/0006-3223(93)90181-C 8218604

[B133] Ustun T. B. Ayuso-Mateos J. L. Chatterji S. Mathers C. Murray C. J. (2004). Global burden of depressive disorders in the year 2000. Br. J. Psychiatry 184, 386–392 10.1192/bjp.184.5.386 15123501

[B134] Vakili K. Pillay S. S. Lafer B. Fava M. Renshaw P. F. Bonello-Cintron C. M. (2000). Hippocampal volume in primary unipolar major depression: a magnetic resonance imaging study. Biol. Psychiatry 47, 1087–1090 10.1016/S0006-3223(99)00296-6 10862809

[B135] Vallee M. Mayo W. Dellu F. Le Moal M. Simon H. Maccari S. (1997). Prenatal stress induces high anxiety and postnatal handling induces low anxiety in adult offspring: correlation with stress-induced corticosterone secretion. J. Neurosci. 17, 2626–2636 9065522 10.1523/JNEUROSCI.17-07-02626.1997 PMC6573515

[B136] van Wingen G. A. van Broekhoven F. Verkes R. J. Petersson K. M. Backstrom T. Buitelaar J. K. (2008a). Progesterone selectively increases amygdala reactivity in women. Mol. Psychiatry 13, 325–333 10.1038/sj.mp.4002030 17579609

[B137] van Wingen G. A. Zylicz S. A. Pieters S. Mattern C. Verkes R. J. Buitelaar J. K. (2009). Testosterone increases amygdala reactivity in middle-aged women to a young adulthood level. Neuropsychopharmacology 34, 539–547 10.1038/npp.2008.2 18235425

[B138] van Wingen G. Mattern C. Verkes R. J. Buitelaar J. Fernandez G. (2008b). Testosterone biases automatic memory processes in women towards potential mates. Neuroimage 43, 114–120 10.1016/j.neuroimage.2008.07.002 18675364

[B139] Veith R. C. Lewis N. Langohr J. I. Murburg M. M. Ashleigh E. A. Castillo S. (1993). Effect of desipramine on cerebrospinal fluid concentrations of corticotropin-releasing factor in human subjects. Psychiatry Res. 46, 1–8 10.1016/0165-1781(93)90002-X 8464952

[B140] Vreeburg S. A. Hoogendijk W. J. van Pelt J. Derijk R. H. Verhagen J. C. van Dyck R. (2009). Major depressive disorder and hypothalamic-pituitary-adrenal axis activity: results from a large cohort study. Arch. Gen. Psychiatry 66, 617–626 10.1001/archgenpsychiatry.2009.50 19487626

[B141] Vythilingam M. Vermetten E. Anderson G. M. Luckenbaugh D. Anderson E. R. Snow J. (2004). Hippocampal volume, memory, and cortisol status in major depressive disorder: effects of treatment. Biol. Psychiatry 56, 101–112 10.1016/j.biopsych.2004.04.002 15231442

[B142] Wang J. Korczykowski M. Rao H. Fan Y. Pluta J. Gur R. C. (2007). Gender difference in neural response to psychological stress. Soc. Cogn. Affect. Neurosci. 2, 227–239 10.1093/scan/nsm018 17873968 PMC1974871

[B143] Weiner C. L. Primeau M. Ehrmann D. A. (2004). Androgens and mood dysfunction in women: comparison of women with polycystic ovarian syndrome to healthy controls. Psychosom. Med. 66, 356–362 10.1097/01.psy.0000127871.46309.fe 15184695

[B144] Weinstock M. (1997). Does prenatal stress impair coping and regulation of hypothalamic-pituitary-adrenal axis? Neurosci. Biobehav. Rev. 21, 1–10 10.1016/S0149-7634(96)00014-0 8994205

[B145] Weinstock M. Matlina E. Maor G. I. Rosen H. McEwen B. S. (1992). Prenatal stress selectively alters the reactivity of the hypothalamic-pituitary adrenal system in the female rat. Brain Res. Mol. Brain Res. 595, 195–200 10.1016/0006-8993(92)91049-K 1467966

[B146] Weniger G. Lange C. Irle E. (2006). Abnormal size of the amygdala predicts impaired emotional memory in major depressive disorder. J. Affect. Disord. 94, 219–229 10.1016/j.jad.2006.04.017 16740316

[B146a] World Health Organization. (2012). Depression, a hidden burden (Fact sheet). Available online at: http://www.who.int/mental_health/advocacy/en/

[B147] Young A. H. Gallagher P. Watson S. Del-Estal D. Owen B. M. Ferrier I. N. (2004). Improvements in neurocognitive function and mood following adjunctive treatment with mifepristone (RU-486) in bipolar disorder. Neuropsychopharmacology 29, 538–545 10.1038/sj.npp.1300471 15127079

[B148] Young E. A. Altemus M. (2004). Puberty, ovarian steroids, and stress. Ann. N.Y. Acad. Sci. 1021, 124–133 10.1196/annals.1308.013 15251881

[B149] Young E. A. Kornstein S. G. Harvey A. T. Wisniewski S. R. Barkin J. Fava M. (2007b). Influences of hormone-based contraception on depressive symptoms in premenopausal women with major depression. Psychoneuroendocrinology 32, 843–853 10.1016/j.psyneuen.2007.05.013 17629629 PMC2100423

[B150] Young E. A. Korszun A. (2002). The hypothalamic-pituitary-gonadal axis in mood disorders. Endocrinol. Metab. Clin. North Am. 31, 63–78 10.1016/S0889-8529(01)00002-0 12055991

[B151] Young E. A. Midgley A. R. Carlson N. E. Brown M. B. (2000). Alteration in the hypothalamic-pituitary-ovarian axis in depressed women. Arch. Gen. Psychiatry 57, 1157–1162 10.1001/archpsyc.57.12.1157 11115329

[B152] Young E. A. Ribeiro S. C. Ye W. (2007a). Sex differences in ACTH pulsatility following metyrapone blockade in patients with major depression. Psychoneuroendocrinology 32, 503–507 10.1016/j.psyneuen.2007.03.003 17462829 PMC1975691

[B153] Zobel A. W. Yassouridis A. Frieboes R. M. Holsboer F. (1999). Prediction of medium-term outcome by cortisol response to the combined dexamethasone-CRH test in patients with remitted depression. Am. J. Psychiatry 156, 949–951 10360139 10.1176/ajp.156.6.949

[B154] Zuloaga D. G. Carbone D. L. Handa R. J. (2012a). Prenatal dexamethasone selectively decreases calretinin expression in the adult female lateral amygdala. Neurosci. Lett. 521, 109–114 10.1016/j.neulet.2012.05.058 22668856 PMC3395764

[B155] Zuloaga D. G. Carbone D. L. Hiroi R. Chong D. L. Handa R. J. (2011). Dexamethasone induces apoptosis in the developing rat amygdala in an age-, region-, and sex-specific manner. Neuroscience 199, 535–547 10.1016/j.neuroscience.2011.09.052 22008524 PMC3237835

[B156] Zuloaga D. G. Carbone D. L. Quihuis A. Hiroi R. Chong D. L. Handa R. J. (2012b). Perinatal dexamethasone-induced alterations in apoptosis within the hippocampus and paraventricular nucleus of the hypothalamus are influenced by age and sex. J. Neurosci. Res. 90, 1403–1412 10.1002/jnr.23026 22388926

